# Spontaneous patella fracture associated with anterior tibial tubercle pseudarthrosis in a revised knee replacement following knee Arthrodesis

**DOI:** 10.1186/1471-2474-14-317

**Published:** 2013-11-06

**Authors:** Alfonso Manzotti, Simone Aldè, Chris Pullen, Pietro Cerveri, Norberto Confalonieri

**Affiliations:** 1 1st Orthopaedic Department, C.T.O. Hospital, Via S. Pertini 21,Via Bignami 1, 20040, Cambiago Milan, Italy; 2 Royal Melbourne Hospital, Grattan Street, Parkville, Victoria Australia; 3 Bioengineering Department, Politecnico di Milano, Milan, Italy

**Keywords:** Knee, Arthrodesis, Arthroplasty, Revision, Complication, Patella

## Abstract

**Background:**

Conversion of a knee arthrodesis to a Total Knee Arthroplasty is an uncommon procedure. Revision Total Knee Arthroplasty in this setting presents the surgeon with a number of challenges including the management of the extensor mechanism and patella.

**Case presentation:**

We describe a unique case of a 69 years old Caucasian man who underwent a revision Total Knee Arthroplasty using a tibial tubercle osteotomy after a previous conversion of a knee arthrodesis without patella resurfacing. Unfortunately 9 months following surgery a tibial tubercle pseudarthrosis and spontaneous patella fracture occurred. Both were managed with open reduction and internal fixation. At 30 months follow-up the tibial tubercle osteotomy had completely consolidated while the patella fracture was still evident but with no signs of further displacement. The patient was completely satisfied with the outcome and had a painless range of knee flexion between 0-95°.

**Conclusions:**

We believe that patients undergoing this type of surgery require careful counseling regarding the risk of complications both during and after surgery despite strong evidence supporting improved functional outcomes.

## Background

Knee arthrodesis is an uncommon salvage operation. The restrictions to everyday life that result from a fused knee can lead to considerable patient dissatisfaction. This has led to patients seeking conversion of the arthrodesis to a total knee arthroplasty (TKA) and several authors underline how this conversion can result in a better functional result than a fused knee [1,2]. No clear guidelines for this procedure are available in literature. Holden et al. [2] recommended that a constrained implant should be used in conversion of a fused knee to a TKA to compensate for the lack of soft tissue stabilizers. Kim et al. [3] proposed that even in the most straightforward cases a posterior stabilized TKA should be used.

All Authors point to a significant rate of complications such as early loosening, soft tissue necrosis and infections following conversion of a knee arthrodesis to a TKA [1,2,4,5]. Henkel et al. [4] reported that 86% of their patients who underwent conversion of a knee arthrodesis to TKA required re-operation with complications including skin necrosis, extensor mechanism contracture, insufficient collateral ligaments, and adhesion/arthrofibrosis. Clemens et al. [5] reported a significant incidence of infection following skin necrosis after this surgery and suggesting an intra-operative gastocnemius transfer and skin graft. Management of the knee extensor mechanism is difficult in these operations and is often complicated by patella baja and knee stiffness [6-9]. A tibial tubercle osteotomy is usually advocated but may result in additional complications including tibial tubercle pseudarthrosis. The risk of tibial tubercle pseudarthrosis has been shown to some extent to be dependent on surgical technique [6-9].

No authors to our knowledge have reported a spontaneous patella fracture following conversion of a knee arthrodesis to a TKA. In the literature, patella fracture following revision TKA has a reported incidence ranging from 0.2% to 21% [10-13]. Ninety percent of these fractures occurred when the patella had been resurfaced (88%) often without specific trauma or significant symptoms [14-18]. Seijas et al. [14] reported 2 cases of atraumatic non-resurfaced patella fracture following a primary TKA in 2009 highlighting that this was an extremely uncommon event. Factors associated with atraumatic patellar fractures include patellar subluxation, improper patellar resection, vascular compromise, component designs and thermal necrosis [13,15]. Even restoration of postoperative flexion in a previous stiff knee has been proposed as a potential cause of fracture [13,15].

Non-operative treatment is advocated for minimally displaced fractures. For displaced fractures open reduction and internal fixation with revision of the patella component, with or without augmentation and patellectomy have been recommended [11,12,15-18]. To the best of our knowledge no report regarding the treatment either of a tibial tubercle pseudarthrosis or of an atraumatic patella fracture after a revision TKA following knee arthrodesis has been published in the literature. The aim of this case report is to illustrate a unique complex case of a patient presenting with these 2 conditions simultaneously.

## Case presentation

**Figure 1:**
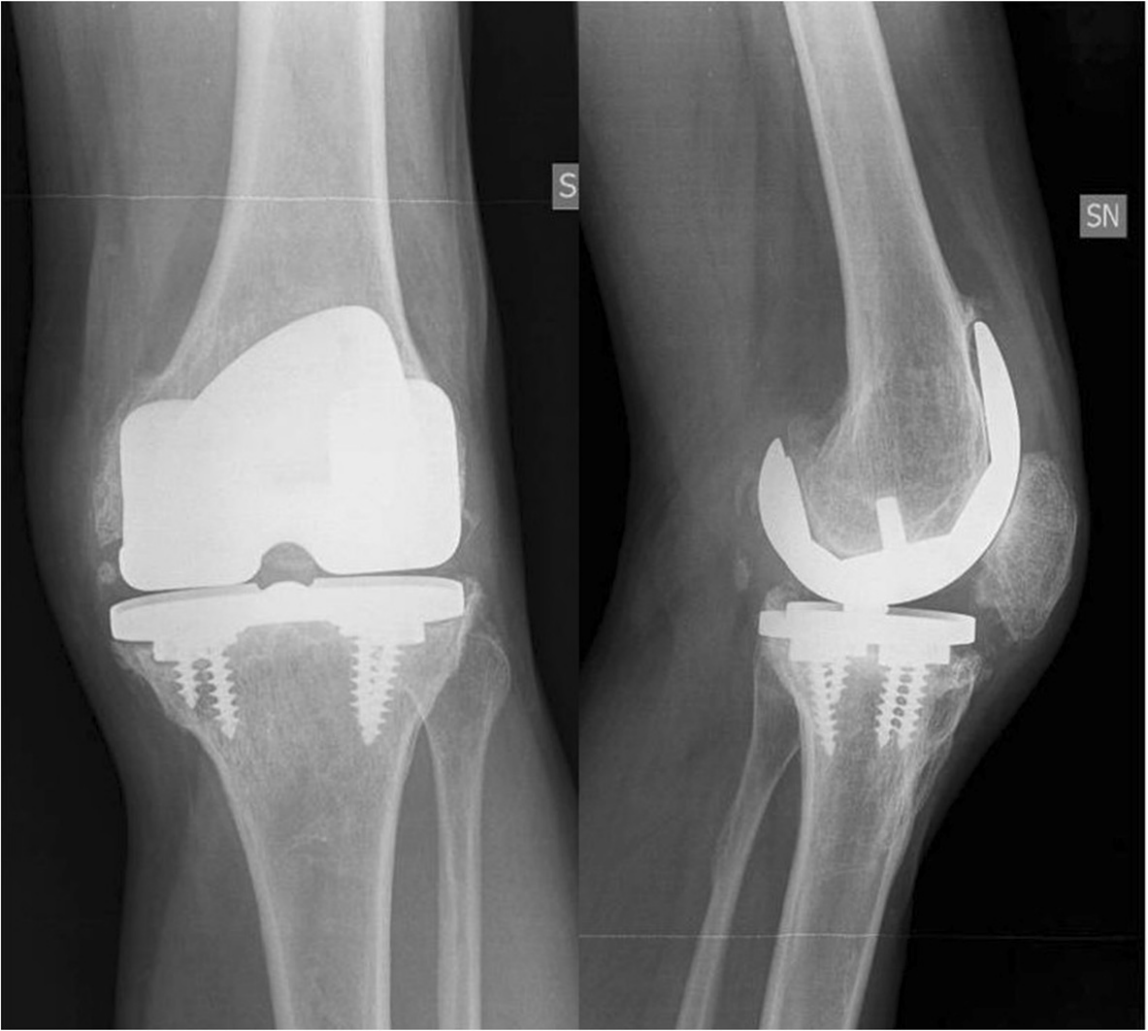
Preoperative radiographs showing the original knee implant following the arthrodesis with clear radiolucency.

**Figure 2:**
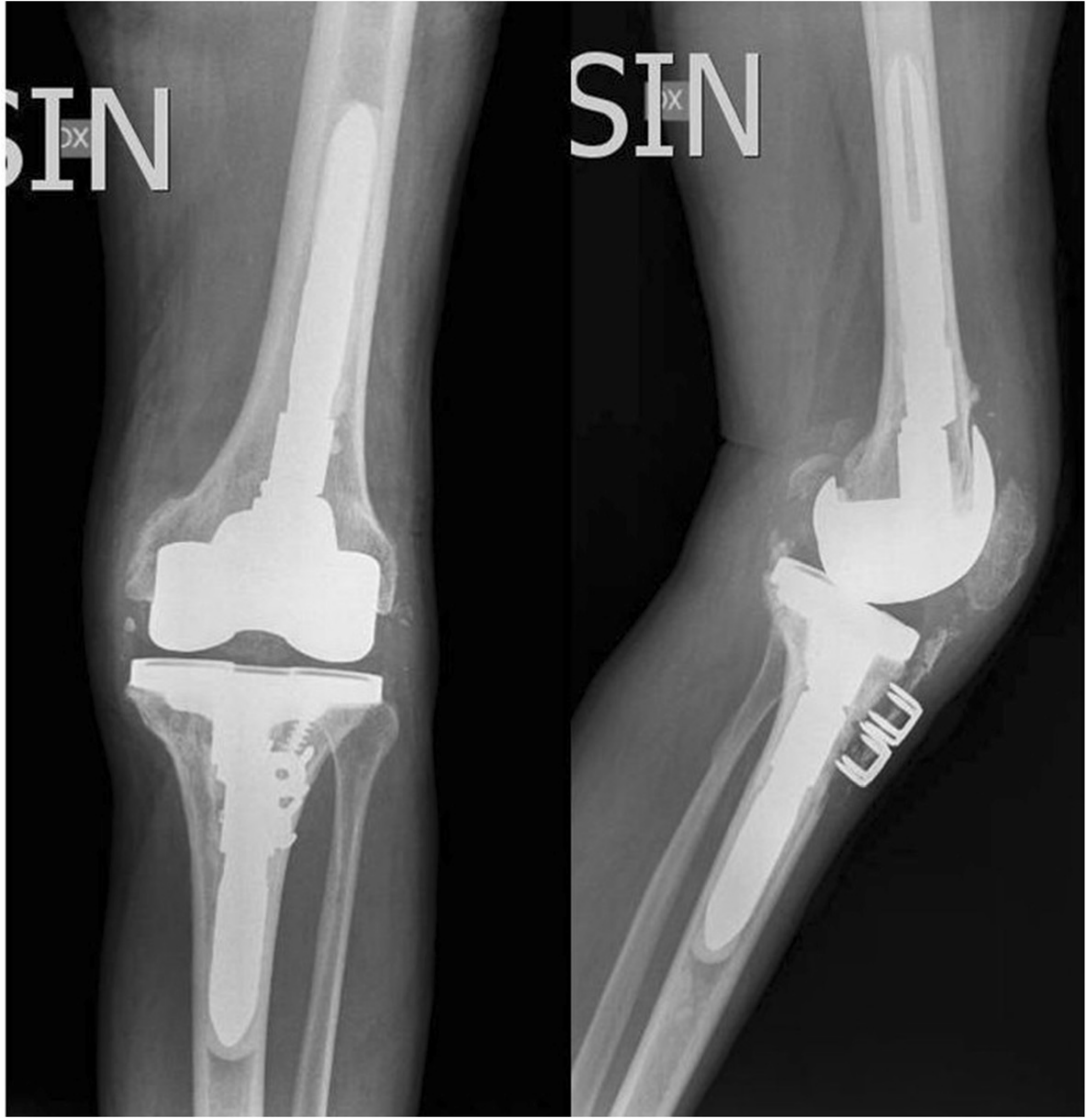
Post-operative radiographs following the TKA revision without resurfacing the patella and 2 staples used to fix the medially detached tibial tubercle.

A 69-year-old healthy male was referred to our department for revision left TKA. He had complained of chronic knee pain and stiffness following conversion of a knee arthrodesis to a TKA. He had initially undergone an open knee arthrodesis 50 years earlier for septic arthritis with a post-operative long rigid knee extension brace for 3 years. Conversion of the knee arthrodesis to a TKA was performed in 1997 at another hospital with a non-cemented cruciate sparing implant. The patella was not resurfaced. In the early post-operative period full knee extension and 75° of active flexion was achieved. The outcome deteriorated after a few years with the patient developing worsening stiffness and increasing pain and for these reasons a TKA revision had already been recommended. At his presentation the left leg was 1 cm shorter than the right. The circumference of the left thigh 5 cm above the patella was 3.5 cm smaller than the right. The left knee was stable with a valgus alignment. All movements of the left knee caused pain. Full extension of the left knee but less than 20° active flexion was seen with a mobile patella. There was no clinical evidence of knee sepsis or effusion. Radiographic evaluation showed the mechanical axis of the left lower limb was 188°. Patella baja and clear signs of loosening of both the femoral and tibial components were evident (Figure 1). After obtaining informed consent in March 2008 the patient underwent to a revision TKA using a cemented semi-constrained prosthesis (Legion, Smith & Nephew, Memphis, Tennessee, USA). At operation a mid-line para-patellar approach was made partially detaching the anterior tibial tuberosity (6 cm long bone block) on the medial side. The osteotomy was subsequently reattached with 2 staples and reasorbable sutures. Intraoperatively polyethylene wear was seen in the lateral femoro-tibial compartment. No signs of active sepsis were seen either on inspection or on microbiological examination of intraoperative specimens. The femoral and tibial components were easily removed without further bone loss leaving in-situ a broken tibial screw. Femoral and tibial stems with offsets were used. An oxinium femoral shield with 25 mm distal wedges was used to reduce the patella baja by lowering the joint line. All components were fixed using antibiotic impregnated cement. The patella was not resurfaced or reduced to avoid weakening the patella bone. Correct patella tracking and range of motion was observed intra-operatively (Figure 2).

**Figure 3:**
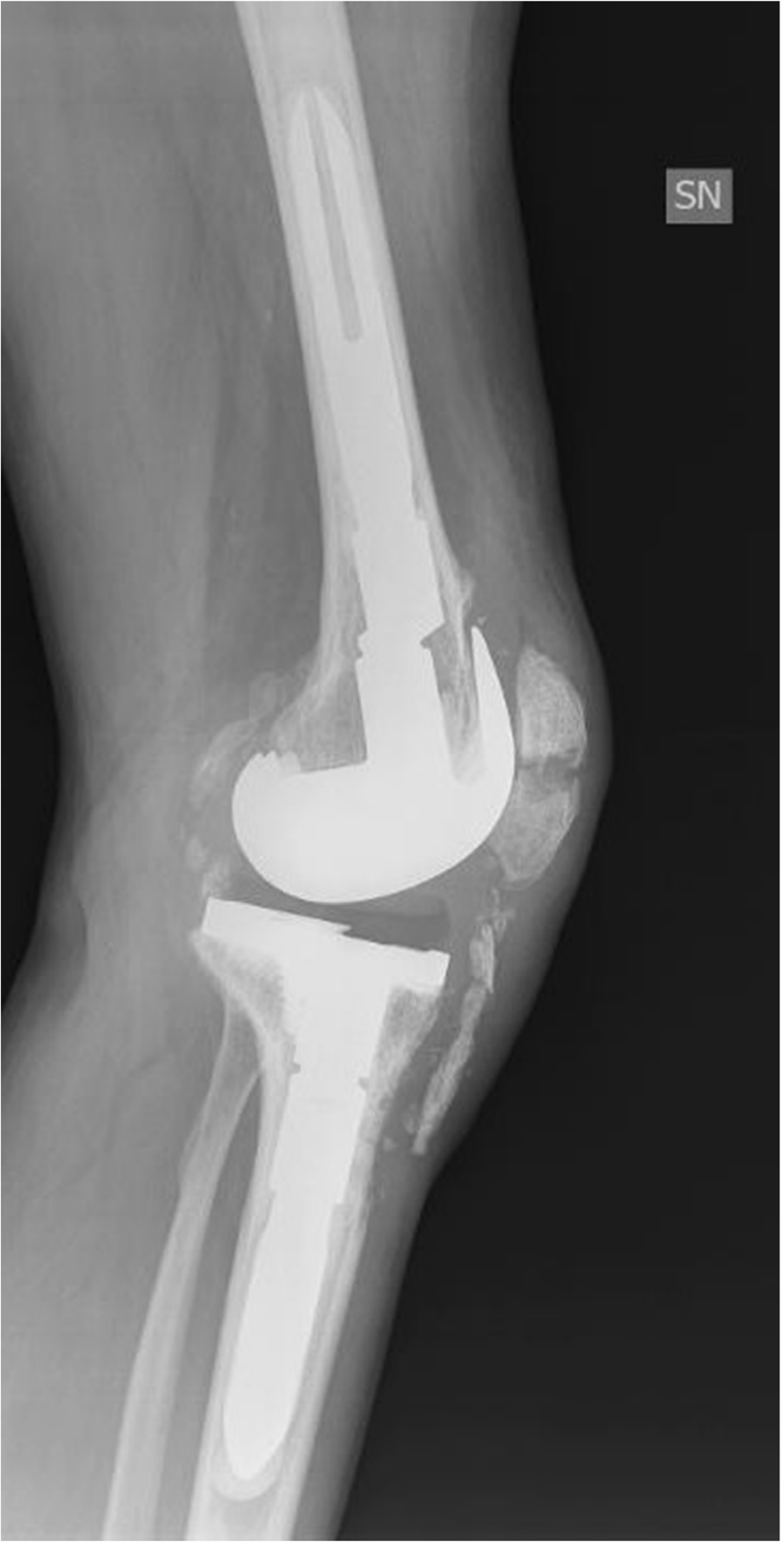
Spontaneous patella fracture 9 months following the revision procedure.

**Figure 4:**
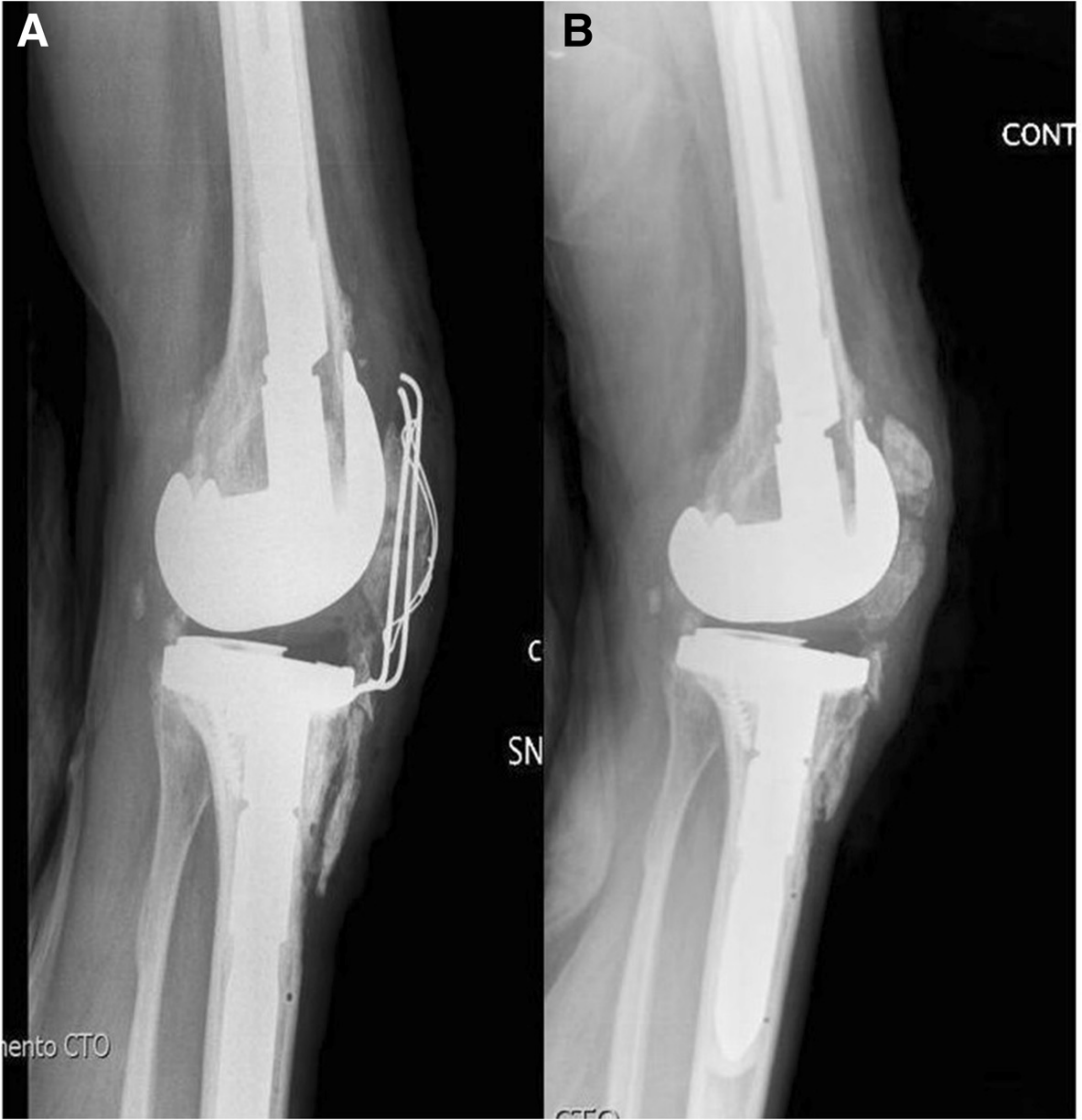
**A-B: Open reduction and internal fixation of the patella fracture and tibial tubercle pseudarthrosis and 36 months follow-up radiographs, showing healing of the tibial tubercle and incomplete patella fracture healing.**

A hinged knee brace was worn post-operatively with flexion limited to 30° for 20 days. Full weight bearing was encouraged as soon as tolerated. After brace removal progressive passive flexion was commenced with 95° of painless flexion achieved at 2 months after surgery. At 2 months post-operatively the patient was able to walk without pain. Despite incomplete healing, the tibia tuberosity staples were removed at 5 months after the revision surgery because of progressive staple loosening under the skin without any influence on the range of motion (0-95°). Nine months after revision knee surgery, following rising from bed and flexing the knee, the patient experienced acute left patella pain. Radiological assessment at that time showed a moderately displaced transverse mid-patellar fracture (Figure 3). The patient was treated with open reduction and internal fixation of the fracture using a tension band and k-wire technique. At the same surgery repeat tibial tubercle fixation was performed again using trans-osseous reabsorbable sutures (Figure 4A). The previous post-operative rehabilitation protocol was used including a hinged knee brace with flexion limited to 30° for 20 days, and full weight bearing as soon as tolerated. Hardware removal was undertaken 9 months after fracture fixation for knee pain on maximal flexion even though the patella fracture had not completely healed. Radiographs at this time suggested fibrous union of the patella fracture. At the 36-month follow-up despite no further radiological sign of fracture union, no sign of fracture displacement was seen and it appeared clinically stable. At this time the tibial tuberosity was clearly consolidated. (Figure 4B). No further displacement of the patella fracture was clinically or radiographically evident during flexion. The patient’s knee was painless with flexion of 0-95°. He was able to maintain a full painless knee extension against gravity and walk without aids. He was completely satisfied with the result. Left thigh atrophy 5 cm above the patella had improved from 3.5 to 2 cm.

## Conclusions

Our case report deals with a healthy male who experienced an anterior tibial pseudarthrosis and a spontaneous patella fracture 9 months after revision TKA in which no resurfacing of the patella was performed. He had 50 years earlier undergone a knee arthrodesis and this was converted to a primary TKA in 1997. Following conversion of the knee arthrodesis to a TKA he achieved 75° of knee flexion for only a short period. His clinical result then progressively deteriorated and at presentation he had painful knee flexion to 20° only. Revision TKA resulted in 0-95° of painless knee flexion at 36 months follow-up. A possible explanation for the poor outcome achieved following the primary TKA may be the use of a cruciate retaining implant which is not usually recommended in conversion of a knee arthrodesis to a TKA [19]. Pseudarthrosis of the tibial tubercle occurred despite only partial detachment probably because of use of too small a bone block and/or unstable fixation. According to the literature the use of staples and reabsorbable sutures may be inadequate fixation of a tibial tubercle osteotomy [4,5]. We considered this was one of the causes of the pseudarthrosis and this has resulted in a change to a more stable fixation with either 4.5 mm canulated screws or metallic cables in all subsequent cases.

Both the tibial tubercle pseudarthrosis and patella fracture were simultaneously managed with open reduction internal fixation despite being minimally displaced. Complete tibial tubercle consolidation was obtained and despite incomplete radiographic patella fracture healing, both excellent knee function and patient satisfaction was achieved. We are uncertain if a non-operative treatment would have achieved a similar result.

In conclusion, we believe that where a revision TKA is required after a previous conversion of a knee arthrodesis, the patient requires careful counseling regarding the high rate of complications both during and after the operation. Furthermore the surgeon should be prepared to face a large number of different potential complications. However, in our patient even a less than perfect result achieved at revision TKA was preferred to the previous stiff painful knee replacement.

### Consent

Written informed consent was obtained from the patient for publication of this case report and any accompanying images. A copy of the written consent is available for review by the Editor of this Journal.

## Abbreviations

TKA: Total knee arthroplasty.

## Competing interests

The authors declare that they have no competing interests.

## Authors’ contributions

AM was the surgeon in chief and drafted the manuscript, NC supervised both the revision surgery and the study helping to draft the manuscript, PC and SA contributed in datas acquisition and helped to draft the manuscript, CP helped to draft the manuscript and supervised the English translation. All authors read and approved the final manuscript.

## Pre-publication history

The pre-publication history for this paper can be accessed here:

http://www.biomedcentral.com/1471-2474/14/317/prepub
